# Application of omics technology to combat the COVID‐19 pandemic

**DOI:** 10.1002/mco2.90

**Published:** 2021-09-16

**Authors:** Jingjing Yang, Yunzheng Yan, Wu Zhong

**Affiliations:** ^1^ National Engineering Research Center for the Emergency Drug Beijing Institute of Pharmacology and Toxicology Beijing China; ^2^ School of Pharmaceutical Sciences Hainan University Haikou Hainan China

**Keywords:** COVID‐19, genomics, metabolomics, proteomics, transcriptomics

## Abstract

As of August 27, 2021, the ongoing pandemic of coronavirus disease 2019 (COVID‐19), caused by severe acute respiratory syndrome coronavirus 2 (SARS‐CoV‐2), has spread to over 220 countries, areas, and territories. Thus far, 214,468,601 confirmed cases, including 4,470,969 deaths, have been reported to the World Health Organization. To combat the COVID‐19 pandemic, multiomics‐based strategies, including genomics, transcriptomics, proteomics, and metabolomics, have been used to study the diagnosis methods, pathogenesis, prognosis, and potential drug targets of COVID‐19. In order to help researchers and clinicians to keep up with the knowledge of COVID‐19, we summarized the most recent progresses reported in omics‐based research papers. This review discusses omics‐based approaches for studying COVID‐19, summarizing newly emerged SARS‐CoV‐2 variants as well as potential diagnostic methods, risk factors, and pathological features of COVID‐19. This review can help researchers and clinicians gain insight into COVID‐19 features, providing direction for future drug development and guidance for clinical treatment, so that patients can receive appropriate treatment as soon as possible to reduce the risk of disease progression.

## Graphical abstract

In the global COVID‐19 pandemic, multiomics‐based strategies have been applied into the surveillance of newly emerged SARS‐CoV‐2 variants and the study of diagnostic methods, risk factors, and pathological features of COVID‐19, which will contribute to the prevention and control of this pandemic.

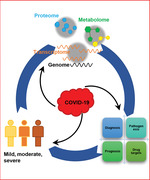

## 1 INTRODUCTION

In 2019, severe acute respiratory syndrome coronavirus 2 (SARS‐CoV‐2), a highly infectious virus related with the coronavirus disease 2019 (COVID‐19), was first identified and spread rapidly across the world, seriously threatening global public health and causing destructive impact on the economy.^1^

During this COVID‐19 pandemic, in order to keep track of the latest epidemic, classical epidemic models, such as susceptible–infected–recovered model and susceptible–infected–recovered–dead model, as well as modified and sophisticated models were employed to estimate daily morbidity and recovery rates, as well as to predict the time and peak of confirmed cases^2^, ^3^, ^4^ As of August 27, 2021, the ongoing COVID‐19 pandemic has spread to over 220 countries, areas, and territories, and 214,468,601 confirmed cases have been reported to World Health Organization, including 4,470,969 deaths.^5^ At present, the United States of America (USA) is deeply affected by the COVID‐19 pandemic, and there is a daily increase of over 100 thousand confirmed cases in the past week.^6^ Meanwhile, this pandemic is spreading at alarming rates in India, Brazil, the Russian Federation, and some Asian countries.^7^, ^8^, ^9^ So far, a variety of candidate drugs, such as Chloroquine phosphate, Remdesivir, and Favipiravir, have been used to explore the effectiveness against SARS‐CoV‐2 infection, whereas no specific drugs for the treatment of SARS‐CoV‐2 infection were generally accepted. Vaccination is another crucial means to control infectious diseases, and there are multiple SARS‐CoV‐2 vaccines approved for marketing currently. However, obstacles, such as rapid mutation of virus, pose huge challenges to the application prospects of vaccines. In view of this, it is urgent to realize in‐depth analysis and understanding of SARS‐CoV‐2 and COVID‐19 so as to achieve the prevention and control of this major epidemic situation.

Omics technology, including genomics, transcriptomics, proteomics, and metabolomics, can achieve high‐throughput detection and analysis of target samples, which has been greatly enriched and developed in recent decades. Without exception, multiomics‐based strategies have been widely used to investigate the pathogenesis, potential drug targets, and diagnostic approaches of COVID‐19.^10^, ^11^, ^12^ In particular, genomic analysis has been applied to identify the mutations of SARS‐CoV‐2 as well as track the emergence of new variants. Transcriptome sequencing has been used to detect changes in SARS‐CoV‐2 gene expression in different biological samples.^13^, ^14^ Proteomics and metabolomics analyses have been conducted to detect and quantify proteins and metabolites, whereas different analytical methods have been used to understand cell behavior after infection with the virus, identify the entry receptors of SARS‐CoV‐2, investigate the pathogenesis of COVID‐19, find promising druggable targets, and facilitate the development of specific drugs and vaccines.^15^, ^16^, ^17^ In this review, we mainly summarize the latest progress in applications of genomics, transcriptomics, proteomics, and metabolomics in COVID‐19 pandemic.

## 2 GENOMICS ANALYSIS OF SARS‐COV‐2

As the COVID‐19 pandemic spread, more and more mutations were detected in newly isolated SARS‐CoV‐2 strains, indicating adaptive viral evolution.^18^ Further, currently licensed vaccines are mainly designed for the original version of S protein; however, if they can still be protective against newly emerging SARS‐CoV‐2 mutant S protein is still dubious.^19^ On this basis, the disclosure of enough SARA‐CoV‐2 genomes for genomic surveillance is of great importance to track the mutations, evolution, and adaptation of SARS‐CoV‐2,^6^, ^20^, ^21^ and series of efforts were made to realize real‐time genomic surveillance.^22^, ^23^

### 2.1 Genomic surveillance of SARS‐CoV‐2

The ongoing evolution of SARS‐CoV‐2 has been the topic of considerable interest as the pandemic spreading globally.^24^ Once a new prevalent variant arises in one country, it will quickly become a threat to neighbors.^25^ Evidence is growing that SARS‐CoV‐2 variants could evade immune responses under the selective pressure triggered by vaccines and previous infections, and new variants are being detected more frequently.^6^ Therefore, the strength and duration of both natural and vaccinal SARS‐CoV‐2 immunity remains will play a central role in shaping the future dynamics of COVID‐19 cases and drowned a global rush to increase genomic surveillance.^9^

As the progression of COVID‐19 pandemic, genomic surveillance has contributed significantly in tracking variants. Series of newly emerging variants are identified for further study, including B.1.1.7 (also called Alpha variant), B.1.351 (also called Beta variant), P.1 (B.1.1.28.1, also called Gamma variant), B.1.167.2 (also called Delta variant), B.1.427/B.1.429 (CAL.20C, also called Epsilon variant), P.2 (B.1.1.28.2, also called Zeta variant), B.1.525 (also called Eta variant), P.3 (B.1.1.28.3, also called Theta variant), B.1.526 (also called Lota variant), B.1.167.1 (also called Kappa variant), C.37 (also called Lambda variant), and some other variants spread in different regions.^26^, ^27^, ^28^, ^29^, ^30^, ^31^ Among them, B.1.1.7 variant, B.1.351 variant, P.1 variant, and B.1.617.2 were designated as variants of concern (VOCs).^32^

The SARS‐CoV‐2 B.1.1.7 variant originated in the UK from late Summer to early Autumn 2020. Recently, it has rapidly spread from southeast England to the whole world (at least 185 countries, territories, and areas), indicating a substantial selective advantage over other currently circulating lineages.^33^, ^34^ Increased transmissibility, risk of hospitalization, and reinfection add urgency to intensive monitoring of B.1.1.7 variant.^19^, ^35^, ^36^ The signature mutation N501Y may be partially responsible for the phenomenon.^37^, ^38^, ^39^

B.1.351, the main lineage circulating widely in South Africa during the second wave of infections, is characterized by eight lineage‐defining mutations in S protein of SARS‐CoV‐2, including K417N, E484K, and N501Y on the RBD, L18F, D80A, D215G, ∆ 242–244 on the NTD and A701V located in S2 region.^6^, ^38^ Since its appearance, the B.1.351 lineage almost completely displaced other lineages. They could be 50% more transmissible than the other circulating variants.^40^ Additionally, the researchers found that B.1.351 was more resistant to convalescent serum and vaccine‐induced sera than other circulating variants in the pandemic.^41^, ^42^, ^43^

The emergence of SARS‐CoV‐2 P.1 variant (B.1.1.28.1) was observed in a surge in severe COVID‐19 case hospitalizations in Brazil in February, 2021. Through whole‐genome sequencing, the P.1 lineage was revealed to be associated with rapid increase of confirmed cases and hospitalization rates.^44^, ^45^ This new P.1 lineage bearing 17 mutations, including 11 mutations on the S protein. These mutations resulted in increased binding to the human angiotensin‐converting enzyme 2 (ACE2) receptor and immunity evasion against multiple neutralizing monoclonal antibodies, convalescent plasma, and vaccinee sera, which will threaten current antibody therapies and lead to increased reinfection rates.^46^, ^47^ P.2 variant (B.1.1.28.2) is a sub‐lineage of B.1.1.28, which distinguished from P.1 for the single mutation E484K on the RBD of S protein. This variant was first reported in Rio de Janeiro, and then spread to the others states of Brazil.^48^, ^49^ P.3 variant (B.1.1.28.3), which carried N501Y and E484K on the RBD of S protein, is another sub‐lineage prevalent in Philippines.^50^, ^51^

The recently emerging variants almost all carried the L452R mutation, including B.1.427/B.1.429 variant, B.1.526 variant, and B.1.167 variant. B.1.427/B.1.429 variant (also CAL.20C), first identified variant bearing a L452R mutation, is the prevalent lineage spread in California, USA, which possesses increased infectivity and resistance of neutralization due to the cooccurrence of L452R, S13I, and W152C mutation.^30^, ^52^, ^53^ B.1.526 variant was identified in New York City in November 2020, accompanied by controversial breakthrough infection.^54^, ^55^, ^56^ SARS‐CoV‐2 B.1.167.1 is one of the circulating variants of India, which had spread worldwide.^57^ B.1.167.2, also known as Delta variant, is first detected in the United Kingdom in April, 2021, and subsequently, the emergence of this variant was traced back to October, 2020 in India. As of August 10, 2021, it has been reported by over 140 countries, territories, and areas around the world.^58^ B.1.167.2 carries T19R, ∆157‐158, L452R, T478K, D614G, P681R, and D950N mutations on the S protein, indicating high transmissibility and breakthrough infection of vaccinated people, as well as increased hospitalized patients.^28^, ^57^, ^59^

Apart from VOCs, series of emerging variants have been monitored for further alerts. A.23.1 is a sub‐lineage of A.23 defined by F157L, V367F, Q613H, and P681R mutations, possessing increased fusion activity and immune evasion ability. In January, 2021, A.23.1 overtook the former circulating variants in Uganda, and spread rapidly to more than 23 countries.^31^ R.1 variants is a lineage first detected in USA and Europe, which rapidly prevailed in Tokyo in March, 2021.^60^ And B.1.525 (also called Eta variant), which charactered by the carrying of signature mutations of VOCs, is first identified in Nigeria and poses incredible risk on unvaccinated population in Africa. Also, the possible resistance to vaccine‐induced immunity will facilitate its worldwide spreading.^61^, ^62^ Lambda variants (C.37), carrying L452Q and T859N, are circulating in Peru during January to April, 2021 and could be responsible for the steep increase of confirmed cases in South America. As reported, L452Q and D614G are considered to increase the transmissibility, and T859N could be responsible for a reduced neutralization by monoclonal antibodies and by convalescent and postvaccination sera (Table 1).^29^

**TABLE 1:** Mutations on the S protein of circulating SARS‐CoV‐2 variants

	Variants	Mutations on S protein	Signature mutations	References
Alpha^a^	B.1.1.7	N501Y, ∆69/70, ∆144/145, A570D, P681H, T716I, S982A, and D1118H	N501Y	^63^, ^64^
Beta^a^	B.1.351	D614G, L18F, D80A, D215G, ∆242‐244, R246I, K417N, E484K, N501Y, and A701V	∆242‐244, R246I, K417N, E484K, N501Y	^42^, ^64^, ^65^
Gamma^a^	P.1 (B.1.1.28.1)	D614G, L18F, T20N, P26S, D138Y, R190S, K417T, E484K, N501Y, H655Y, and T1027I	K417T, E484K, N501Y	^45^, ^46^, ^64^
Delta^a^	B.1.167.2	T19R, G142D, L452R, T478K, D614G, P681R, and D950N	L452R, T478K	^28^, ^66^, ^67^
Epsilon	B.1.427/B.1.429 (CAL.20C)	S13I, W152C, L452R, and D614G	L452R, S13I, W152C	^30^, ^52^, ^53^
Zeta	P.2 (B.1.1.28.2)	D614G, L18F, T20N, P26S, D138Y, R190S, E484K, H655Y, T1027I, and V1176F	E484K, V1176F	^48^
Eta^b^	B.1.525	Q52R, A67V, ∆H69/V70, ∆Y144/145, E484K, D614G, Q677H, and F888L	∆69/70, ∆145, E484K, Q677H	^61^
Theta	P.3 (B.1.1.28.3)	D614G, ∆LGV141‐143, E484K, N501Y, P681H, E1092K, H1101Y and V1176F	E484K, N501Y, P681H	^50^, ^51^
Lota^b^	B.1.526	L5F, T95I, D253G, D614G, A701N, and S477N or E484K	L5F, T95I, D253G, S477N or E484K	^56^
Kappa^b^	B.1.167.1	D614G, G142D, E154K, L452R, E484Q, P681R, Q1071H, and H1101D	L452R, E484Q	^61^, ^67^, ^68^
Lambda^b^	C.37	G75V, T76I, R246N, Δ247‐253, L452Q, F490S, D614G, and T859N	L452Q, T859N	^29^

^a^ VOCs, variants of concern.

^b^ VOI, variants of interest.

### 2.2 Notable mutations of SARS‐CoV‐2

**FIGURE 1:**
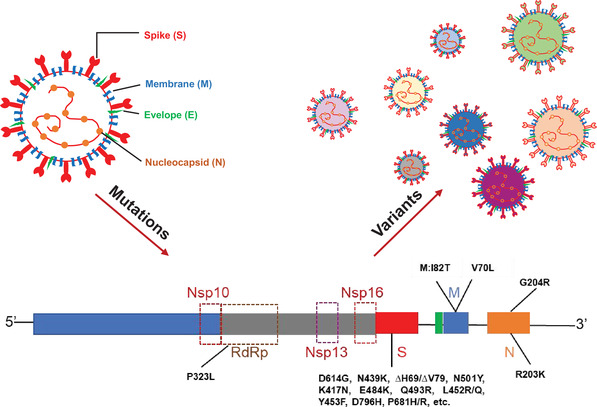
Notable mutations carried in circulating variants. S, spike protein; M, membrane protein; N, nucleocapsid protein; RdRp, RNA‐dependent RNA polymerase; Nsp, nonstructural protein; Nsp13, helicase; Nsp10–Nsp16, methyltransferase complex

The emergence of new variants is driven by the heritable mutations of SARS‐CoV‐2 (Figure 1). As reported, SARS‐CoV‐2 can utilize all ACE2 proteins, except mouse ACE2, as cell entry receptors.^69^ And the highly variable spike (S) protein of SARS‐CoV‐2 is responsible for virus–cell interaction. S protein is composed of S1 and S2 subunits; virus attachment and entry are directly mediated by the receptor binging domain (RBD) of S1, whereas the fusion peptide (FP) of S2 facilitates membrane fusion.^26^, ^70^ During the SARS‐CoV‐2 epidemic, the mutations of S protein are rapidly expanding, while most mutations are either lost, or occasionally fixed, at the point of transmission, with minimal persistence of shared diversity.^24^, ^71^

At the early stage of COVID‐19 pandemic, some mutations have existed in the epidemic strains, including H49Y on the S1 N‐terminal domain (NTD) from China, G476S on the RBD from Washington, USA and S943P on FP from Belgium.^72^ As the COVID‐19 pandemic going on, more and more mutations are identified, including some prominent mutations that spread globally. D614G (G614) mutation on the C‐terminal domain 2 (CT2) leads to an open conformational state of S protein, which will facilitate the binging of RBD to ACE2, indicating that strains with D614G mutation in the S protein possess stronger virulence and transmission ability.^73^, ^74^ Also, D614G mutations showed a selective advantage of higher viral loads, younger patient age, and reinfection among circulating variants.^75^, ^76^, ^77^

N439K mutation on the receptor binding motif (RBM) of S protein can enhance the binding affinity of S protein to ACE2 receptor moderately and can lead to resistance against several neutralizing monoclonal antibodies and some polyclonal sera from persons recovered from infection.^78^, ^79^ ∆H69/∆V70 deletion in the S1 NTD of the S protein is responsible for the compensation of immune evasion‐induced infectivity defect in prevalent circulating variants, whereas the NTD ∆242–244 deletion, which has similar functional consequences, is particular in certain strains.^37^, ^80^, ^81^ N501Y, K417N/K, and E484K are key contact residues in RBD.^43^ Their mutations, especially the combined mutation of N501Y and E484K, can particularly enhance the binding affinity of RBD to hACE2. Also, some researchers have found N501Y mutation may promote the binding affinity of RBD to mouse ACE2s, posing a risk of intermediate transmission of SARS‐CoV‐2.^82^ Furthermore, N501Y and K417N can only decrease the neutralization ability of certain vaccines, whereas E484K can cause widespread escape from monoclonal antibodies and convalescent plasma neutralization. Further, Δ242–244 will be additive to the resistance of immune barrier as well as T95I.^41^, ^65^, ^83^, ^84^, ^85^, ^86^ Q493R is emerged after the treatment of E484K mutant SARS‐CoV‐2 infections with bamlanivimab/etesevimab, indicating the incidence of potential drug resistance.^87^ L452R and Y453F, two naturally occurred substitutions in RBM of S protein, can enhance the interaction of SARS‐CoV‐2 and ACE2 as well as be resistance of cellular immunity. L452R/Q mutation is almost carried in all newly emerging variants, the emergence of L452R/Q not only increase viral infectivity and fusogenicity, but also decrease the sensitivity of certain variants to neutralizing antibodies.^88^, ^89^ In certain variants, L452R mutation is occurred with W152C and S13I, leading to increased infectivity.^52^

In chronic infection, D796H substitution in the S2 subunit of S protein appeared to be responsible for the resistance of neutralizing antibodies accompanied by decreased infectivity; however, when combined with ∆H69/∆V70 mutation, this defect can be compensated.^90^ Mutation P681H/R is immediately adjacent to the furin cleavage site in S protein, which can enhance the fusion activity of the SARS‐CoV‐2 and promote S1/S2 cleavage by the cellular furin protease, so as to benefit the proliferation of SARS‐CoV‐2.^31^, ^36^, ^37^ Besides, novel m6A methylation locus in the S protein of SARS‐CoV‐2 is also detected to change the virulence and transmission capacity of the virus, which call for comprehensive sequencing of circulating variants.^91^

Aside from S protein, RNA‐dependent RNA polymerase (RdRp), methyltransferase complex (Nsp10–Nsp16), and helicase (Nsp13) can also be promising drug targets of SARS‐CoV‐2 infection. However, the emergence of mutations poses a challenge on the development of virus‐targeted therapies. By now, silent mutation and composition‐related mutations are identified in the RdRp gene of SARS‐CoV‐2, arisen a risk on possible drug‐resistance to clinical used RdRp‐targeted drugs.^92^ Moreover, studies have reported mutations in methyltransferase complex (Nsp10–Nsp16) and helicase (Nsp13) of SARS‐CoV‐2, which can facilitate host–pathogen interaction.^93^, ^94^ Membrane (M) protein participates many virus–cell interactions. M: I82T and V70L mutations, which located in the glucose transport region of M protein, can enhance the uptake of glucose during viral replication, and these mutations will be resulted in SARS‐CoV‐2 infection in younger people.^95^ Nucleocapsid (N) protein of SARS‐CoV‐2 has a region associated with nuclear localization signals, which raised another concern of emerging mutations. As reported, amphimutation of R203K&G204R can enhance the transportation of NP to nucleus and contribute to high case fatality rates.^96^

Moving forward, long‐term and continuous tracking of emerging variants and the functional consequences of mutations will help researchers and politicians to formulate more appliable strategies for the prevention and control of COVID‐19 pandemic, including vaccination strategies.

## 3 APPLICATION OF TRANSCRIPTOMICS IN COVID‐19 PANDEMIC

**FIGURE 2:**
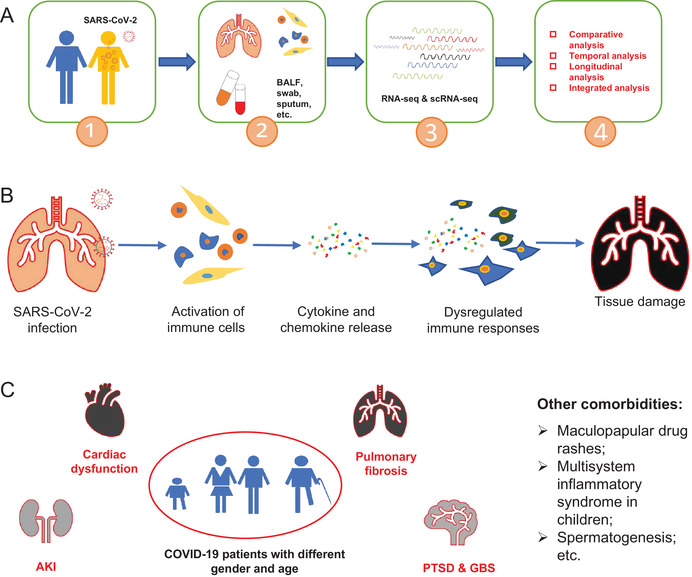
SARS‐CoV‐2 infection induced tissue damage and comorbidities. (A) Workflow of transcriptomic analysis of clinical samples collected from COVID‐19 patients; (B) mechanisms of SARS‐CoV‐2 infection induced tissue damage; (C) SARS‐CoV‐2 infection correlated comorbidities

Being an RNA virus, the life cycle of SARS‐CoV‐2 is highly dependent on the machinery of the host cell. Therefore, once infected by SARS‐CoV‐2, series of changes occur in the host cells, especially in their transcriptomes. Transcriptomic analysis is useful for investigating the cellular mechanisms underlying risk factors, pathogenesis, and potential drugs targets of SARS‐CoV‐2 infection. Further, the application of transcriptomic analysis in various clinical samples will be helpful to elucidate the mechanisms of COVID‐19‐associated tissue damage and comorbidities (Figure 2).

### 3.1 Dysregulated immune responses in COVID‐19 patients

Many studies have confirmed that attenuated antiviral responses and inappropriate inflammatory responses exist in COVID‐19 patients, including persistently enriched T cells and monocytes.^97^, ^98^ SARS‐CoV‐2 infects alveolar macrophages to induce the production of T cell chemoattractants, and lead to the activated T cells. Then, the activated T cells began to produce interferon‐γ (IFN‐γ), and IFN‐γ will promote the release of inflammatory cytokines, including T cell chemoattractants, from alveolar macrophages in turn, and resulted in further T cell activation.^98^ During this loop, clonally expanded cluster of differentiation 8 (CD8+) T cells and an increased ratio of CD8+ effector T cells to effector memory T cell characterized severe disease, while circulating follicular helper T cells accompanied mild disease.^99^ Besides, elevated inflammatory response will enhance monocytic infiltration into lungs.^100^ Uncommitted CD34+ hematopoietic stem/progenitor cells were primed toward megakaryopoiesis, accompanied by expanded megakaryocyte‐committed progenitors and increased chemokine/cytokine release.^99^ Nonclassical CD16+ monocytes, which is characterized by decreased antigen presentation, suppressed monocyte response to early antiviral interferon signals and deficient lymphocyte expression of cytotoxicity genes. Also, IFN‐driven early B cell activation was reduced.^101^, ^102^ Therefore, overexpressed chemokines and cytokines, as well as low levels of IFN‐I and IFN‐III accompanied moderate interferon‐stimulated genes response, are proposed to drive the progression of COVID‐19.^103^, ^104^, ^105^

Heterogeneous activation of coagulation and fibrinolytic pathways are present in early stage of COVID‐19 and will persist into its late stage.^106^ SARS‐CoV‐2 infection causes endothelial disruption and vascular thrombosis in lungs, which are induced by macrophage infiltrating, as well as the upregulation of complement, platelet activation, thrombosis, and proinflammatory factors‐related genes.^107^ The peak expression of these dysregulated genes was accompanied by respiratory failure as well as chemokine‐dominant hypercytokinemia.^104^, ^108^, ^109^ Subsequently, hypoxia inducting factor system (HIF) was detected, along with the arisen of oxidative phosphorylation (OXPHOS), reactive oxygen species (ROS) and heme‐related metabolic pathways mediated mitochondrial dysfunctions, which will further contribute to the amplification of immune dysfunction as well as related systematic hypoxia injury.^110^, ^111^, ^112^, ^113^

### 3.2 Risk factors of severe COVID‐19

It has been reported that ACE2 is the receptor of SARS‐CoV‐2. During viral entry, the S protein is primed by transmembrane protease serine 2 (TMPRSS2). To determine the susceptible factors of SARS‐CoV‐2 infection, series of experiments were conducted and multiple risk factors of severe COVID‐19 were demonstrated.

#### 3.2.1 Susceptible factors of severe COVID‐19

The analysis and reanalysis of single‐cell transcriptomic data specialized on ACE2 and TMPRSS2 found coexpression of ACE2 and TMPRSS2 in pulmonary and extrapulmonary tissues, including maternal–fetal interface, salivary glands, and the granulosum of the skin and so on, posing a risk of systematic tissue damage, as well as indicating the possibilities of vertical transmission and contact transmission.^114^, ^115^, ^116^, ^117^

In moderately or critically ill patients with COVID‐19, ACE2 and TMPRSS2 expression in epithelial cells of lung was increased in response to Type 2 inflammation‐related interleukin‐13 (IL‐13) and IFN signals. Afterward, increased *ACE2* and *TMPRSS2* expression contributed to clinical inflammatory lung injury and respiratory failure. In this procedure, series of inflammation‐related pathways, including Toll‐like receptor 4 (TLR4), C‐C chemokine receptor type 1 (CCR1), CCR5, C‐X‐C chemokine receptor type 6 (CXCR6), mammalian target of rapamycin (mTOR), mitogen‐activated protein kinase (MAPK)/MAPKK/protein kinase B (also known as AKT), inhibitor of nuclear factor κB kinase/nuclear factor κB (NF‐κB), and ferroptosis pathways, were involved.^14^, ^118^, ^119^, ^120^, ^121^, ^122^, ^123^

SARS‐CoV‐2 infection induces hyperactivation of the extrinsic blood coagulation cascade and the suppression of the plasminogen activation system in epithelial cells,^124^ which poses higher risk for diverse coagulopathies in the lung and distal organ systems of COVID‐19 patients. Tissue factor, the key regulator of extrinsic coagulation cascade signaling, could be the most promising drug targets for COVID‐19‐associated coagulopathies, whereas coagulation factor VWF (von Willebrand factor) and ADAMTS13 (a disintegrin and metalloproteinase with a thrombospondin type 1 motif, member 13) may be related to the incidence of severe COVID‐19.^125^, ^126^ The expression of microtubule‐associated proteins 1A/1B light chain 3B (LC3B) and (p62/SQSTM1) p62, both of which depend on lysosome for degradation, could also predict the emergence of moderate‐to‐severe disease in COVID‐19 patients requiring hospitalization for supplemental oxygen therapy.^127^

Aside from the underlying risks, many acquired risk factors are also observed in SARS‐CoV‐2 infection. The higher expression of ACE2 and TMPRSS2 in individuals who smoked and those with lung cancer, posing relatively higher risk of SAR‐CoV‐2 infection.^128^ As well as a fivefold increase of ACE2 expression was also observed in the heart tissues obtained from patients with obstructive hypertrophic cardiomyopathy.^129^ Besides, in the population of COVID‐19 patients with chronic lung injuries, AT2 cells exhibit preexisting dysregulation of viral infection associated genes, including verified and putative entry receptors and priming proteases (ACE2 and putative BSG, NPR1, HSPA5, as entry receptors; and TMPRSS2, CTSL, or FURIN, as priming proteases), which will facilitate SARS‐CoV‐2 infection.^130^

Besides, COVID‐19 patients could also be distinguished by age and gender. Compared with male and elderly patients, female and children possess higher expression levels of immune modulation‐correlated genes and will experience relatively lower disease severity.^131^, ^132^ Further, reprogrammed immune cell landscape and upregulated expression of susceptibility genes were uncovered in the elderly COVID‐19 patients. Among them, receptor‐interacting serine/threonine‐protein kinase 1 (RIPK1) could be a potential target for drug repurposing in elderly population.^133^, ^134^

#### 3.2.2 Immune microenvironment

The severity of COVID‐19 may depend on the immune microenvironment. In asymptomatic or mild COVID‐19 patients, lower proportion of CD169+ expressing monocytes and correlated proinflammatory cytokines, as well as more counts of mature neutrophils, early bystander CD8+ T cell and plasmablast responses and higher levels of growth factors were detected, suggesting that asymptomatic patients mount less proinflammatory and more protective immune responses against SARS‐CoV‐2, and no prolonged immunological activation would exist.^110^, ^135^, ^136^ Hyperactivation of dendritic cells (DCs), CD14+ monocytes, and megakaryocytes progenitor cells/platelets and reduction of naive CD4+ T lymphocytes were detected in patients with severe COVID‐19, along with proinflammatory monocyte‐derived macrophages enrichment.^137^, ^138^ In addition, the hospitalized patients in critical condition possess increased proportions of cytotoxic follicular helper cells and cytotoxic T helper cells, along with natural killer cells (NKs) deletion induced disruption of CD8+ T cell exhaustion than nonhospitalized patients.^137^, ^139^ Mucosa‐associated invariant T (MAIT) cells can function as innate‐like sensors and mediators of antiviral responses. Dysregulated gene expression of MAIT cells could be associated with poor clinical outcome.^140^ Besides, plasma B‐cell activity and calprotectin were higher in critical COVID‐19, whereas most transcripts related to immune functions were reduced, particularly in B cells.^141^

### 3.3 COVID‐19‐associated tissue damage and comorbidities

SARS‐CoV‐2 infection‐induced systematic tissue damage was marked by inflammation and coagulopathy in blood and tissues.^142^, ^143^ In the lungs of fatal COVID‐19 patients, dysregulated genes were mostly associated with dysregulated activation of granulocyte and complement, lymphocyte differentiation and certain T cell activation, as well as correlated pulmonary fibrosis.^12^, ^144^, ^145^ Overactive immune responses‐related genes resulted in chemokines (such as CXCL1 and CXCL8) induced neutrophil pulmonary infiltration. The abnormal activation of neutrophils, characterized by the enrichment of CD177, alarmin S100 A8/A9/A12, is mediated by the TLR4 pathway, will further contribute to the amplification of inflammatory responses, including activated CXCR2 pathway, in turn. This loop will prolong uncontrolled pathological damage in severe COVID‐19 patients, especially in lung.^12^, ^146^, ^147^, ^148^, ^149^ Otherwise, the genes associated with B cell activation, human leukocyte antigen class DR low monocytes differentiation, neutrophil precursors‐correlated emergency myelopoiesis were also distinguished.^150^, ^151^, ^152^, ^153^

In the postmortem human brain tissue of COVID‐19 patients, monocytes and macrophages infiltrate to choroid plexus across the blood–brain barrier and lead to IRF8‐, ATF5‐, SPI1‐, and TAL1‐mediated activation of microglia inflammatory responses, including cellular activation, mobility, and phagocytosis.^154^ Peripheral T cells can infiltrate to the parenchyma, and astrocyte cluster is marked by established inflammation and astrogliosis, lead to significant dysregulation of neurotransmission and synaptic organization.^155^ In the frontal cortex tissue of COVID‐19 patients, downregulation of genes associated to HIF was observed, which may inhibit the capacity of defense system during infection and oxygen deprivation, showing that hypoxia is also marked in the brain of COVID‐19 patients.^113^

COVID‐19 causes cardiac dysfunction in up to 25% of diagnosed patients, independent of disease severity.^156^, ^157^ Cardiac damage is not only associated with disease mortality,^158^, ^159^ but also leads to a long‐term suffering of cardiac sequelae from COVID‐19.^160^ However, its pathogenesis still needs further study. As reported, phospholipase A2 group VII (PLA2G7), a well‐known cardiovascular disease biomarker, was predominantly expressed by proinflammatory macrophages in lungs at the early stage. However, with the progression of COVID‑19, serum PLA2G7 was also elevated, and posing a risk on cardiovascular system of the COVID‐19 patients.^161^ In the cardiomyocytes of died patients with COVID‐19, multiple genes associated with nuclear disruption and myofibrillar fragmentation, particularly sarcomeric fragmentation, were upregulated.^89^ Besides, in primate cardiopulmonary aging models, IL‐7 accumulated in aged cardiopulmonary tissues and induced ACE2 expression in human vascular endothelial cells in an NF‐κB‐dependent manner, leading to systemic inflammation and compromised virus defense of aging cardiopulmonary.^162^ Taken together, these findings provide an insight into the mechanism of cardiac pathology of COVID‐19, and they may help guide the development of efficacious antiviral and cardioprotective therapies in patients with COVID‐19.^157^

During to the systematic tissue damage in individuals with COVID‐19, various comorbidities are observed in this population. Transcriptomic analysis conducted in the male reproductive system revealed that during the high expression levels of ACE2 in testis, SARS‐CoV‐2 infection‐correlated spermatogenesis damage was observed.^163^, ^164^ Acute kidney injury usually occurs in COVID‐19 patients, accompanied by podocytes and proximal straight tubule cells damage.^165^ Maculopapular drug rashes are associated with hyperactivation of monotypes/macrophages and highly cytotoxic CD8+ T cells in severely ill COVID‐ 19 patients. These cutaneous findings are possibly initiated by or exacerbated by a robust systemic COVID‐19‐induced immune response.^166^ Multisystem inflammatory syndrome in children (MIS‐C) is a new COVID‐19‐related disease mediated by enhanced autoreactivity. In the individuals with MIS‐C, IL‐15‐driven NK cell exhaustion derangement resulted in downregulation of NK cells as well as disrupted CD8+ T cell exhaustion and led to sustained inflammatory environment and severe or even fatal T cell immunopathology.^137^ T‐box transcription factor 21, a central coordinator of exhausted CD8+ T cell differentiation, as well as suppressor of cytokine signaling 1 (SOCS1), a negative regulator of type I and II interferons, may be the promising therapeutic target candidate for MIS‐C.^167^, ^168^ People recovered from COVID‐19 may develop posttraumatic stress disorder.^169^ Augmented T helper 17 cell differentiation and cytokine response might be partially responsible COVID‐19‐associated central nervous system dysregulation, including Guillain‐Barré syndrome.^170^ SARS‐CoV‐2 infection is associated with an increased rate of ischemic stroke and intracerebral hemorrhage mediated by complement cascade. Namely, upregulation of complement component C3 is triggered by SARS‐CoV‐2 infection; C3a amplifies the inflammatory signaling of brain, whereas C3b leads to cellular destruction.^171^ Viral sepsis is also detected in COVID‐19 patients.^172^

## 4 APPLICATION OF PROTEOMICS IN THE EMERGING PANDEMIC

**FIGURE 3:**
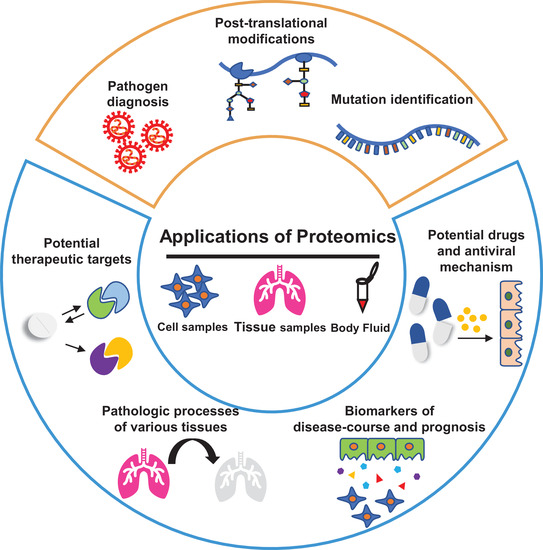
Overview of application of proteomics in COVID‐19 pandemic. The yellow box and blue box represent the application of proteome in the fields of viral detection and monitoring the diversity of host protein levels, respectively

Nowadays, proteomics has been widely used to study and develop therapies of different diseases. In the COVID‐19 pandemic, the application of proteomics can be roughly divided into two aspects: one is directly applied in virus characterization, including pathogen diagnosis, mutation identification, posttranslational modifications, and so on; the other is for monitoring the impacts of infection on host cells at the protein level, including discovery of potential therapeutic targets, understanding the pathologic processes and the immunogenicity, revealing the antiviral mechanism of drugs, as well as detection of biomarkers for disease course and prognosis (Figure 3).

### 4.1 Protein‐based COVID‐19 diagnosis

The development of sensitive and specific SARS‐CoV‐2 detection tool is of utmost priority for the control of its transmission, and early diagnosis of infection will help clinicians administer timely intervention to prevent possible disease progression. At present, the most widely used SARS‐CoV‐2 diagnosis method is RT‐PCR, which is also supposed to be a gold standard^173^; however, viruses in some samples, such as urine, cannot be detected by RT‐PCR, and the accuracy of detection by this method may be affected by various factors, including substitutions and deletions. Aside from RT‐PCR, enzyme‐linked immunosorbent assay is also a popular tool to detect SARS‐CoV‐2 infection. The limitation of this method is that it takes up to 3 weeks for body's immune system to generate antibodies against foreign viral particles.^174^ In addition, conventional techniques for studying IgG and IgM responses in patients can only detect a single target protein in one test. Hence, new diagnosis methods should be developed to supplement the detection of COVID‐19.

Recently, mass spectrometry (MS)‐based proteomic methods with rapid and sensitive superiority have been applied for SARS‐CoV‐2 diagnostic testing. Targeted proteomic analysis was conducted to identify candidate viral peptides targets for SARS‐CoV‐2 detection.^175^ Jiang et al.^176^ constructed a protein‐based microarray for high‐throughput characterization of proteome‐wide antibody responses to aid the identification of potential diagnostic and therapeutic targets of COVID‐19. Also, MS‐based approaches were used to detect SARS‐CoV‐2 N protein in nasopharyngeal epithelial swabs, respiratory tract samples, and gargle solutions with high sensitivity.^177^, ^178^, ^179^, ^180^, ^181^ Collectively, clinical proteomics has become a potential complementary approach for the diagnosis of COVID‐19 by directly detecting viral peptides or proteins. Although these studies have confirmed the feasibility of MS‐based SARS‐CoV‐2 detection strategies, there are some limitations to such approaches, especially regarding the preparation of clinical samples and the baseline of detection, which merits further investigation.^182^, ^183^

### 4.2 Proteome‐associated disease course and prognosis factors of COVID‐19

Identification of disease courses of COVID‐19 is essential to implement preventive measures and personalized intervention, especially in critically patients. Researchers have carried out a number of proteomic studies on different samples to find valuable biomarkers that can accurately predict the COVID‐19 disease trajectories.

By analyzing plasma proteomics of COVID‐19 patients, researchers found that some plasma proteins could be used as biomarkers to predict the disease severity. Haljasmägi et al.^184^ conducted a longitudinal proteomic profiling in terms of blood inflammation markers, antibodies, and plasma proteins in COVID‐19 patients requiring intensive care unit (ICU) admission or not. The results indicated that selective inflammatory markers, such as IL‑6 and CXCL10, as well as monocyte‑attracting C‐C motif ligand 2 (CCL2), CCL7, and CCL8 were significantly evident in ICU cohorts.^184^ In many studies, it has been claimed that IL‐6 has correlation with the severity of COVID‐19, and cytokines related with IL‐6‐mediated proinflammatory signaling are highlighted as biomarkers of critical COVID‐19.^185^, ^186^, ^187^ In addition, there are certain proteomic analysis studies of exosomes and plasma samples, and found that a variety of cytokines including CRP, lactate dehydrogenase (LDH), procalcitonin (PCT), SAA, angiotensinogen (AGT), IL‐12, PTX3, IGLV3‐19, BNC2, CKAP4, and so on are remarkably upregulated or downregulated during the disease progression, which could serve as potential predictors of progression and mortality, although some cytokines are nonspecific and still need further confirmation.^188^, ^189^, ^190^, ^191^, ^192^, ^193^, ^194^ Compared with nonsevere COVID‐19 patients, dysregulation of a variety of apolipoproteins (APOA1, APOA2, APOC1, etc.) in critically ill patients was discovered based on proteomic profiling, although the relationship between these proteins and the disease process needs further explanation.^189^, ^195^, ^196^ Neutrophil activation is the first line of defense against pathogens infection. It can recruit inflammatory mediators to accumulate at the site of infection and even cause cytokine storms when out of control. Many studies have showed that neutrophils are closely associated with the progression of COVID‐19 and may be a considerable target for clinical prediction and therapeutic intervention.^190^, ^197^, ^198^ Factors involved in neutrophil activation, such IL‐8 and resistin, were regarded as the most potent discriminators of critical illness, and it plays a central role in the pathogenesis of severe COVID‐19.^199^, ^200^ Apart from blood, urine samples can also be used to predict the COVID‐19 disease course. For instance, by using capillary electrophoresis‐MS‐based proteome analysis, Wendt et al.^201^ found urinary peptides significantly associated with SARS‐CoV‐2 infection and may be a valuable biomarker to assess and predict the severity of COVID‐19 disease course. Also, Ni et al.^202^ revealed that ACE2 is detectable by MS‐based proteomic approaches in urinary samples, and further verified the feasibility of urinary samples to predict disease severity.

In face of the long‐term recovery of the prognosis of patients infected by SARS‐CoV‐2, Doykov et al.^203^ used targeted proteomic technology to analyze a cohort of serum samples, and the results indicated that even after SARS‐CoV‐2 infections have subsided for a considerable time, biochemical and inflammatory pathways can remain perturbed long. Considering that, it is necessary to identify some valuable prognostic biomarkers to evaluate the therapeutic efficacy and monitor the recovery of COVID‐19 patients. Also noteworthy, prognostic markers and predictors used to classify COVID‐19 severity have a considerable overlap, and in‐depth characterization of these factors should be developed for further applications.^204^

### 4.3 Potential therapeutic targets of COVID‐19 treatment

Investigating the pathogenesis and discovering the potential therapeutic targets of COVID‐19 are critical for the healthcare systems to provide correct interventions. Recently, based on proteomics technology, researchers have performed various attempts and discovered many emerging therapeutic targets. These studies can be roughly divided into three strategies: one is to screen antiviral targets at the cellular level, which is based on the use of human cell lines expressing SARS‐CoV‐2 proteins to explore potential interacting proteins; the other is to directly study the proteomics of various tissue samples from COVID‐19 patients and construct the protein–protein interaction network; the third is to integrate, summarize, and analyze the published proteomic data for revealing more targets with high efficiency and low toxicity.

In a pioneering study on COVID‐19, Gordon et al.^205^ expressed 26 SARS‐CoV‐2 proteins in human cells, which were used for affinity‐purification MS; this study identified 332 high‐confidence protein–protein interactions between SARS‐CoV‐2 and human proteins, and more than 66 of 332 proteins were identified as druggable targets. Based on this work, Acharya et al.^206^ demonstrated that BRD2 is a potential target for development of therapeutics against SARS‐CoV‐2 in vitro. In another study, using proximity proteomics, Meyers et al.^207^ generated a compendium of 2422 human proteins vicinal to 17 SARS‐CoV‐2 viral proteins, which provide insights into the pathogenicity and potential targets of SARS‐CoV‐2. Stukalov et al.^208^ performed a proteomics analysis of SARS‐CoV‐2‐infected ACE2‐expressing A549 cells and revealed perturbation of many antiviral pathways; moreover, the production of ephrin‐B1, polymerase (RNA) II (DNA directed) polypeptide B, thymidylate synthase, and dihydrofolate reductase showed a ubiquitination‐dependent decrease. Furthermore, the ubiquitination changes that occur in both the virus and the Vero E6 cells during SARS‐CoV‐2 infection were identified and quantified and laid a foundation for understanding the relationship between ubiquitination and viral pathogenesis as well as the identification of potential therapeutic targets.^209^ Appelberg et al.^210^ performed an integrative proteo‐transcriptomic analysis of SARS‐CoV‐2‐infected Huh 7 cells and identified tyrosine kinase ErbB, HIF‐1, mTOR, and TNF signaling as potential antiviral targets; and they also clarified and confirmed a Akt/mTOR/HIF‐1 axis. Bojkova et al.^15^ performed quantitative mePROD proteomics analysis on the translatome and proteome, and the results indicated that SARS‐CoV‐2 infection may interfere with different pathways in the host cells, including translation, proteostasis, glycolysis, splicing, and nucleotide synthesis pathways; in addition, the antiviral activities of corresponding inhibitors were verified. The results of bioinformatics analysis of proteomics showed that APOA1, amyloid precursor protein (APP), epidermal growth factor (EGF), complement protein C3 and other targets are also closely related to SARS‐CoV‐2, which could be the valuable diagnostic and therapeutic targets. Significantly, APOA1, as the protein complex seed, was identified as the key differentially expressed protein in different proteomics studies.^195^, ^196^, ^211^

Viral RNA‐binding proteins in the host play a central role in viral replication cycle. Considering that, researchers systematically analyzed viral RNA–protein interactions in cells and revealed that multiple host and viral RNA‐binding proteins are involved in SARS‐CoV‐2 infection.^212^ For positive‐strand RNA viruses, 5′ and 3′ UTRs of viral RNA are associated with its replication and have become a research hotspot recently. Verma et al.^213^ generated RNA–protein–protein interaction network and demonstrated that Lamp2a, an interaction partner of SARS‐CoV‐2 5′ UTRs, can effectively affect the replication in SARS‐CoV‐2‐infected cells. In another study, host proteins G3BP1 and DDX3X were identified as the interacting protein of SARS‐CoV‐2 nucleoprotein and acted on viral RNA life cycle.^213^

Multiorgan proteomic landscape was generated based on autopsy samples from seven organs of 19 COVID‐19 patients. The outcomes of this study uncovered multiple biological and pathological processes involved in COVID‐19, which offer a unique perspective for understanding the pathogenesis and provide novel insights into potential targets for the treatment of SARS‐CoV‐2 infection.^214^ Leng et al.^215^ conducted a quantitative proteomic analysis of fresh lung tissues and identified molecular features of expiratory dyspnea, coagulation disorder, immune activation, and extracellular matrix imbalance. Besides, they also found that the production of chemokines and cytokines, as well as lymphoid organogenesis were regulated by noncanonical NF‐κB/NFKB2 pathway, representing a potential antiviral target.^215^ In another study, by using an integrated quantitative proteomics and phosphoproteomics approach, the proteomic changes in the liver were detected. The results not only provided insight into pathological features caused by SARS‐CoV‐2, but also presented 202 potential therapeutic drug targets, which may be developed as countermeasures against COVID‐19 liver damage.^216^

In addition to perform direct proteomic analysis of SARS‐CoV‐2 infected samples, researchers also reanalyzed published proteomics data. Bock and Ortea^217^ conducted impact pathways analysis and network analysis of currently available proteomics data and verified the importance of inflammatory responses, as well as uncovered the alteration of proteins related to chromosome segregation during mitosis. Moreover, Feng et al.^16^ reanalyzed publicly available proteomics data and identified ubiquitous bromodomain‐containing protein 4, RIPK1, and tissue‐unique receptor expression‐enhancing protein 5 as promising drug targets.

## 5 APPLICATION OF METABOLOMICS IN THE EMERGING COVID‐19 PANDEMIC

**FIGURE 4:**
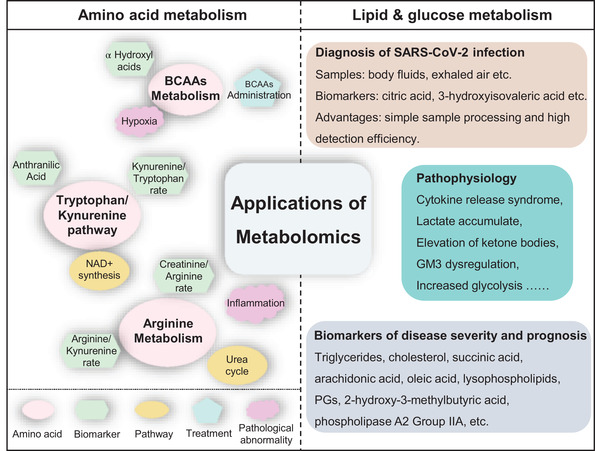
Application of metabolomics in COVID‐19 pandemic. Left column: partial representative amino acid metabolic disturbance; right column: typical application of lipid metabolism and glucose metabolism in COVID‐19 patients

Metabolomics studies focus on the analysis of metabolites perturbations induced by diseases and infection. As direct signatures of disease onset and infection, metabolites can help researchers quickly determine pathogenesis, potential therapeutic targets, as well as cell or tissue damage, and it can also be used as biomarkers to monitor disease progression. At present, metabolomics, including amino acid metabolism, glucose metabolism, lipid metabolism, purine and pyrimidine metabolism, and so on, have been widely applied in the emerging COVID‐19 pandemic. Herein, we summarize the application of metabolomics in identifying pathogenic mechanisms, discovering antiviral targets, and exploring biomarkers from three aspects, namely amino acid metabolism, glucose metabolism, and lipid metabolism (Figure 4).

### 5.1 COVID‐19 associated with amino acid metabolism

Multiple studies have shown that most amino acid metabolism systems, including branched chain amino acids (BCAAs), aromatic amino acids, gluconeogenic amino acids, and so on, are vulnerable to SARS‐CoV‐2 infection. Here, we mainly review the research progress of several amino acids that are more tightly associated with COVID‐19.

Aside from tryptophan/5‐hydroxytryptamine pathway, tryptophan can be metabolized to nicotinamide adenine dinucleotide (NAD) via Kynurenine pathway. Of note, Kynurenine pathway was found to be susceptible to SARS‐CoV‐2 infection and its metabolite, anthranilic acid, could be used as a potentially prognostic biomarker for the evolution of COVID‐19.^218^ Similar findings are also found in other independent experiments. When exploring the changes in the serum metabolites of COVID‐19 patients, Thomas et al.^219^ revealed that tryptophan metabolism via the kynurenine pathway, which is correlated with IL‐6 levels, is altered. Blasco et al.^220^ performed plasma metabolome analysis of COVID‐19 patients at different time points and revealed that metabolites can not only serve as diagnostic biomarkers but also predict the evolution of this disease; moreover, they highlighted the roles of tryptophan–nicotinamide pathway and cytosine in inflammatory signals. As the rate‐limiting step of kynurenine pathway, indole 2,3‐dioxygenase (IDO1) can regulate inflammation and immunization, and the kynurenine/tryptophan ratio, as the presentation of IDO1 activity, may be used as a predictor of disease severity.^219^

Moreover, Shen et al.^196^ collected the sera of COVID‐19 patients to conduct metabolomic analysis and observed the accumulation of 11 steroid hormones that contribute to increased disturbance of NAD+ synthesis via kynurenine pathway as well as macrophage modulation. NAD coenzymes play a significant role in both viral replication and host cell homeostasis. Based on this, Heer et al.^221^ conducted a targeted NAD metabolome analysis and found that the availability of NAD is a key rate‐limiting step of antiviral activities of noncanonical poly‐ADP‐ribose polymerase isozyme; intriguingly, nutritional and pharmacological administration of NAD may enhance the innate immunity to coronaviruses.

Arginine can regulate the activation of host immune cells and resist pathogens infection. Multiple metabolites involved in arginine metabolism also showed abnormal levels in COVID‐19 patients.^196^, ^219^, ^220^ Fraser et al.^222^ performed metabolomics profiling of critically ill COVID‐19 patients admitted to ICU to identify potential diagnostic or prognostic biomarkers in blood and found increased kynurenine as well as decreased arginine, sarcosine, and lysophosphatidylcholines levels; further, it was confirmed that the arginine/kynurenine ratio can accurately determine the COVID‐19 status, whereas the creatinine/arginine ratio can accurately predict COVID‐19‐associated mortality.^222^ Arginine participates in the urea cycle and helps maintain nitrogen balance in the body. However, the levels of arginine in COVID‐19 patients were dramatically reduced, suggesting metabolic reprogramming of urea cycle occurred, and it may cause pathological abnormalities.^223^ In some other studies, arginine metabolism has also been found to be related to inflammatory cytokines and fatal outcomes in COVID‐19 patients.^224^, ^225^ Hypoxia caused by SARS‐CoV‐2 infection can inhibit the OXPHOS of mitochondria and led to a modified BCAA metabolism, which is closely related to the adverse clinical outcomes of severe COVID‐19 patients.^226^ BCAAs administration was recommended as a strategy to maintain NAD balance in COVID‐19 critical patients.

### 5.2 COVID‐19 associated with lipid metabolism and glucose metabolism

Lipid metabolism has a close association with glucose metabolism, and certain metabolites of the two pathways are mutually interconverted. SARS‐CoV‐2‐induced metabolic reprogramming, including lipid metabolism, glycolysis, and tricarboxylic acid (TCA) cycle, provide a comprehensive molecular view of the pathophysiology of COVID‐19. For instance, lipid participates in every process of viral propagation and invasion, and a remarkable alternation of lipid, lipid mediators, and related metabolic pathways was observed in COVID‐19 patients.

#### 5.2.1 Diagnosis of SARS‐CoV‐2 infection

Compared with proteomics, the pretreatment of lipid samples is simpler and the detection efficiency is higher, which helps to achieve rapid determination of SARS‐CoV‐2 infection and mirror the severity of COVID‐19.^227^ To establish a rapid diagnostic approach for SARS‐CoV‐2, De Silva et al.^228^ employed a Teslin^®^ substrate in paper spray MS (PS‐MS) to determine the metabolomic biomarkers of SAR‐CoV‐2 infection within 60 s of analysis time, and further identified 11 metabolites for integrative analysis using symptomatic PCR. Using metabolomics strategy, multiple metabolites in the exhaled air of COVID‐19 patients were considered to have the potential for SARS‐CoV‐2 detection and as biomarkers of disease deterioration.^229^ In another study, Delafiori et al.^230^ combined metabolomics and machine learning to create an expeditious platform for the specific diagnosis of SARS‐CoV‐2 infection. Moreover, this method also provides molecular information about the disease pathophysiology and helps to identify prognostic markers and treatment targets.^230^

#### 5.2.2 Biomarkers for COVID‐19 surveillance

Through metabolomics analysis of serum samples of COVID‐19 patients, it was found that multiple metabolites related to glucose metabolism and lipid metabolism can be used as biomarkers for COVID‐19 disease monitoring. Shi et al.^231^ revealed that a combination of metabolites including d‐fructose, citric acid, and 2‐palmitoyl‐glycerol can be applied to distinguish SARS‐CoV‐2‐infected patients from healthy people; and 2‐hydroxy‐3‐methylbutyric acid, 3‐hydroxybutyric acid, cholesterol, succinic acid, l‐ornithine, oleic acid, and palmitelaidic acid can accurately predict the possibility of severe conditions.^231^ In an untargeted metabolomics study of COVID‐19 plasma, Barberis et al.^232^ found that triglycerides and free fatty acids(arachidonic acid and oleic acid) positively correlated with the severity of COVID‐19; circulating lipids (phosphatidylcholine 14:0_22:6, phosphatidylcholine 16:1_22:6, and phosphatidylethanolamine 18:1_20:4) and multiple metabolites, such as 2‐hydroxy‐3‐methylbutyric acid, 2,3,4‐trihydroxybutyric acid, and 3‐hydroxyisovaleric acid, can act as biomarkers in the detection of COVID‐19 infection.^232^ Besides, the changes of triglycerides in patients with different severity were specifically explored and 11 triglycerides were selected to accurately differentiate severe conditions of COVID‐19.^233^ Phospholipase A_2_ is a class of enzymes that hydrolyze phospholipids to produce fatty acids and lysophospholipids, and specifically, phospholipase A_2_ Group IIA can be used to distinguish SARS‐CoV‐2 infection or not and the gravity of diseases.^234^ In COVID‐19 patients, most glycerophospholipids decrease, whereas lysophospholipids, arachidonic acid, and oleic acid were opposite.^232^, ^235^ As the critical characteristic of dyslipidemia of COVID‐19 patients, dypolipidemia, including low‐density lipoprotein cholesterol, high‐density lipoprotein cholesterol and cholesterol, was observed in several studies of lipid profiles on COVID‐19 patients, which are generally decreased with the gravity of the disease.^236^, ^237^

#### 5.2.3 Disturbance of lipid metabolism and glucose metabolism

SARS‐CoV‐2 infection is closely associate with body's physiological functions and metabolic regulation. Cytokine release syndrome (CRS) is the main cause of multiorgan injury and fatal outcome in critical COVID‐19 patients. Xiao et al.^224^ analyzed the serum samples of COVID‐19 patients and found that reprogrammed host metabolism was closely related to proinflammatory cytokines/chemokines and proposed that metabolic regulation may be a potential strategy for treating fatal CRS. The therapeutic strategy based on this insight provides a novel direction for the treatment of fatal CRS induced by SARS‐CoV‐2 infection.

Using untargeted and targeted metabolomic method, Jia et al.^223^ revealed that lactate accumulates abundantly in severe patients, and even in recovery group whose SARS‐CoV‐2 PCR test is negative, providing an insight of a serious disturbance in energy metabolism. Meanwhile, metabolites in the TCA cycle, including glucose, lactate, and pyruvate, exhibit abnormal levels in the course of COVID‐19. All of these results suggest that TCA cycle and related metabolic pathways have close association with the pathological processes of COVID‐19 diseases.^223^ A metabolomic analysis of red blood cells from COVID‐19 patients revealed markedly increased glycolysis due to the higher protein levels of phosphofructokinase and some other rate‐limiting enzymes or the significantly decreased levels of phosphoglucomutase‐2‐like 1 and glyceraldehyde 3‐phosphate dehydrogenase.^238^

With respect to arachidonic acid and its lipid mediators (prostaglandins, thromboxane, lipoxin, and leukotrienes), there is a close correlation with COVID‐19 pathophysiology. Importantly, arachidonic acid and other unsaturated fatty acids (such as eicosapentaenoic acid and docosahexaenoic acid [DHA]), as well as their metabolites not only can be regarded as potential antiviral agents, but also play a role in inflammation regulation in severe COVID‐19.^239^ It was reported that lipid mediators are related to the course of COVID‐19 disease severity. For instance, PGs (PGE2, PGD2, and PGF2a) and RvE3 exhibited a negative correlation with increasing of disease deterioration. Lipid mediators have immune‐regulatory function and are closely associated with inflammatory progression, which provides a basis for the mechanism study of immuno‐lipidomic disproportion in critical COVID‐19 patients.^240^

The elevation of ketone bodies (acetoacetic acid, 3‐hydroxybutyric acid, and acetone) was reported in the serum of COVID‐19 patients, indicating a pathological condition in which the liver has an abnormal capacity to oxidize acetyl‐CoA.^241^ Sphingolipids are involved in various biological processes, such as apoptosis and inflammatory responses. The levels of sphingolipids are susceptible to viral infection, although there are different trends in COVID‐19 patients.^196^, ^233^, ^235^ Song et al.^235^ utilized a combination of targeted and untargeted MS to analyze the plasma lipidome and metabolome in mildly, moderately, and severely ill COVID‐19 patients and found that monosialodihexosyl gangliosides (GM3s) were increasingly enriched in the exosomes of COVID‐19 patients with greater disease deterioration, and that GM3 dysregulation was one of the pathogenesis mechanisms of COVID‐19. In severe patients, the levels of different ceramides and glycosylceramides were obviously increased and decreased, respectively. And this may be associated with abnormal cardiovascular and pulmonary function of COVID‐19 patients.^233^ For patients during convalescence, metabonomic analysis can monitor the recovery of patients. For example, metabolic changes of plasma samples, including the decrease of palmitic acid and the increase of docosapentaenoic acid as well as DHA, indicated that COVID‐19 patients are undergoing liver repair.^242^

## 6 CONCLUSIONS

Omics technologies are remarkable research approaches for the development of rapid responses to emerging or reemerging infectious diseases. In case of the COVID‐19 pandemic, omics techniques help researchers and clinicians to comprehensively understand and recognize the SARS‐CoV‐2 virus and correlated COVID‐19. Based on the surveillance of the SARS‐CoV‐2 sequences, mutations and variants mediated potential uncontrolled outbreak are closely monitored. And transcriptomics, proteomics, and metabolomics work together to elucidate the underneath pathogenesis of COVID‐19, which will facilitate the identification of biomarkers, risk factors, and drug targets of SARS‐CoV‐2 infection, and realize the early diagnosis and treatment of COVID‐19, so as to fight against the worldwide spread COVID‐19 pandemic.

## CONFLICT OF INTEREST

The authors declare no conflict of interest.

## ETHICS APPROVAL

Not applicable.

## AUTHOR CONTRIBUTION

Jingjing Yang and Yunzheng Yan collected the literatures and drafted manuscript. Wu Zhong revised and edited the manuscript. All authors approved this manuscript for publication.

## Data Availability

Not applicable.
